# Outside versus inside the home: Tensions between fathers’ work and parenting responsibilities in Tanzania

**DOI:** 10.1371/journal.pone.0341670

**Published:** 2026-01-29

**Authors:** Sein Kim, Juliet K. McCann, Damas Joachim, Mary Kabati, Joshua Jeong

**Affiliations:** 1 Department of Global Health and Population, Harvard T.H. Chan School of Public Health, Boston, Massachusetts, United States of America; 2 Hubert Department of Global Health, Rollins School of Public Health, Emory University, Atlanta, Georgia, United States of America; 3 Tanzania Home Economics Organization, Mwanza, Tanzania; Caleb University, NIGERIA

## Abstract

Globally, mothers are disproportionately responsible for childcare, while fathers are predominantly viewed as breadwinners. Although recent studies reveal that fathers are becoming more engaged in parenting, little is known about the broader contexts surrounding how fathers allocate their time between parenting, income-generating work, and other daily activities. The objective of this study was to characterize the typical daily schedules from morning to evening among fathers with young children under aged 2 years. Additionally, we aimed to identify the perceived barriers and facilitators influencing fathers’ time spent on parenting. We conducted 60 in-depth interviews (29 fathers and 23 mothers and 8 community stakeholders) and 9 focus group discussions (3 with fathers only, 2 with mothers only, and 4 mixed parent groups) across 4 purposively sampled, peri-urban communities in Mwanza, Tanzania. Among a subsample of 14 fathers, we revisited and conducted follow up in-depth interviews to further obtain retrospective hourly accounts of fathers’ daily time use. Data were analyzed using both deductive and inductive approaches. Overall, fathers spent most daytime hours out of the home engaging in work-related activities, and evenings were often a time when some fathers socialized with peers. Approximately half of fathers reported some involvement in parenting, childcare, or household chores. However, these activities were generally brief and concentrated at specific times of day, such as early mornings before fathers left for work, during lunchtime when men returned home, or on weekends when men spent more time at home. Key barriers to fathers’ time allocation to parenting included patriarchal gender norms that frame childcare as primarily women’s responsibility, poverty, and work-related stress. Facilitators of fathers’ time towards parenting included greater autonomy over work schedules and positive attitudes valuing fathers’ nurturing care roles. These findings provide important insights into fathers’ daily routines in this context and the social, cultural, and structural factors that enable or constrain their engagement in parenting. By illuminating men’s time use patterns, this study offers evidence to inform the design of parenting programs that are more responsive to fathers’ schedules and constraints and ultimately promote greater father engagement in childcare.

## Introduction

Globally, fathers’ primary roles have often centered on being breadwinners and authoritarians within the household, responsible for financial provision, decision-making [1], exerting power, and maintaining strict discipline [2,3]. While these roles have dominated much discourse, fathers have also been acknowledged as direct caregivers, with their involvement in activities such as carrying and holding varying across context [4–6]. Mothers, on the other hand, have been expected to be much more engaged with overseeing childcare and household responsibilities than fathers, despite also having access to other allomothering resources from other family members [7,8]. In recent decades, however, these gender roles have evolved across low-and middle-income countries (LMICs) due to various socio-economic factors such as increasing maternal labor force participation, shifting gender norms, and changing family structures driven by urban migration [2,9,10]. Contemporary fatherhood in LMICs is encompassing a broader range of expectations and responsibilities, extending beyond mere financial provisions to additionally include more shared time between fathers and their children.

Given the extended caregiving role of fathers, father-inclusive interventions have been growing and have generally shown positive impacts in maternal, couples, and child outcomes [11–14]. For example, an experimental study in Rwanda using small group discussion aimed at changing gender norms found reduction in intimate partner violence, increased use of health services among women, and more equitable decision-making among couples [15]. While the implementation of father-inclusive interventions has been growing in recent years, several interventions have encountered challenges in effectively recruiting, engaging, and retaining fathers [16–18]. For example, overall attendance rates were higher for mothers than fathers in couples-based parenting program in rural Uganda [19]. In another example of a responsive parenting intervention in Western Kenya, 49% of fathers did not attend any sessions despite program efforts to engage them [20]. Similarly, a parenting program in Jordan found that only 20% of fathers participated in the program [21].

Time constraints are a primary barrier to both fathers’ engagement in parenting practices and relatedly their participation in community-based parenting programs. Social and gender norms have largely shaped expectations around fatherhood in terms of the ability to provide for the family, driving men to spend most of their time out of the home engaged in income-generating activities [22–24]. The absolute amount of time fathers spend on parenting remains consistently lower than that of mothers [20,25,26]; for example, a cross-sectional study in Zanzibar, Tanzania, reported that less than half of fathers had played with their child in the past three days at the time of the survey [27]. At the same time, recent studies have suggested that social norms are evolving to value more nurturing forms of fatherhood, which in turn are translating to men’s prioritization of time spent with their children [23,28]. Although several studies have characterized how fathers generally spend most of their days out of the home for work [17,29], few studies have contextualized fathers’ time use for work in relation to other specific activities in their daily routines. Failure to consider the full range of paternal roles, responsibilities, and time use can lead to misinterpretations that fathers are uninterested or unwilling to engage in parenting activities. Thus, investigating the perceived tensions and trade-offs in fathers’ time allocations inside versus outside the home can provide a more holistic understanding of their overall time use and the opportunities for their engagement in parenting practices and programs.

In this study, we examine how fathers allocate their time during a typical day, including the diversity of activities that fathers engage from morning to evening, where and with whom they commonly spend their time, and the barriers and facilitators of fathers’ involvement in parenting in Mwanza, Tanzania. We draw upon qualitative data collected with fathers, mother, and other community members to triangulate our understanding about fathers’ roles, demands, and circumstances. Through this analysis, we aim to illuminate the social context surrounding fathers’ time use to inform programmatic strategies that can increase fathers’ time allocated to parenting.

## Methods

### Study setting

This qualitative study was conducted in the Ilemela district, in Mwanza Region of Tanzania, which is a peri-urban setting located on the southern shores of Lake Victoria. Ilemela district was specifically selected due to the strong existing relationships between the local study partner, Tanzania Home Economics Organization- Mwanza (TAHEA-Mwanza), and community leaders in the district as established through previous collaborations and partnerships. Based on 2022 Demographic Health Survey, 24% of men and 21% of women aged 15–49 have completed secondary education or higher in Mwanza. Men are most frequently employed in skilled manual work, followed by agriculture and unskilled manual labor. Women are most employed in sales and in agriculture. About 40% women work seasonally and are more likely than men to engage in unpaid or family-owned work. Regarding household composition, 70% of household heads in Tanzania are male. Among children under 18, about a half of them live with both parents. Despite some progress in early childhood development in Tanzania, only 48% of children aged 24–59 months are developmentally on track in health, learning, and psychosocial well-being in Mwanza region [30,31].

### Study design and procedures

Four communities were purposively selected via a stratified design in collaboration with TAHEA-Mwanza to capture two coastal communities and two inland communities. This stratification allowed us to explore variation in fatherhood and family-caregiving experiences across the primary paternal occupations of fishing and farming [32]. Data were collected as a part of a broader study that used a descriptive phenomenological qualitative design to understand the parting roles, couples’ relationships, and mental health of fathers in Mwanza, Tanzania [31]. For this study, we conducted a secondary analysis of these data focused on understanding fathers’ time use based on qualitative data from primarily fathers but also mothers and community stakeholders.

To sample study participants, local leaders within each of these locations constructed a list of all caregivers in their communities who were eligible for this study. Caregivers were eligible for participation if they met the following criteria: adult biological parent who was aged 18–65 years; had a child younger than 2 years of age; was in a relationship with the child’s other biological parent; and resided in the same house as partner and child at some point during the past month. Using the list of eligible caregivers, the study team randomly recruited mothers and fathers for in-depth interviews (IDIs) and focus group discussions (FGDs). Focus groups discussions were conducted with mothers alone, with fathers alone, and with mothers and fathers together in mixed groups. Mother-only and father-only FGDs conducted separately were used to create a comfortable environment for participants to speak freely, particularly on sensitive topics such as parenting challenges, gender norms, and household decision-making. On the other hand, mixed-parent FGDs encouraged dialogue between men and women and allowed exploration on how mothers and fathers discussed topics such as gender roles, parenting responsibilities, and time use in one another’s presence. Mixed-group discussions were intended to uncover dynamics, areas of consensus, or differing views that might not emerge in single-gender groups and to triangulate findings across participant groups. Notably, we did not find substantial differences in thematic content between the mixed-parent FGDs and the single-gender FGDs. Within each community, we additionally sampled the community health worker and community leaders for IDIs. We used purposive sampling to select these community stakeholders based on their influence or focal role within the community; these individuals were either elected or hired by the local government or by community members. Their regular interaction with families enabled us to explore perspectives on fathers’ parenting practices beyond the household and individual levels [31].

### Data collection

Data were collected from June 8th to June 29^th^, 2022 by a team of five research assistants bilingual in English and Kiswahili. Research assistants received a 7-day training on qualitative research methods and interviewing skills. IDIs and FGDs were conducted in Kiswahili in a private setting that was centrally located in the participant’s community. Research assistants followed a semi-structured topic guide, which were adapted for each stakeholder group (caregivers and community stakeholders) and data collection type (IDIs and FGDs). The guides used for caregivers (both fathers and mothers) focused on their own experiences as parents, while questions were adapted for the community stakeholder guide (used for both community health workers and community leaders), to elicit their perspectives on community norms and perceptions of parents’ experiences in their communities.

We gathered data on fathers’ time use from two distinct sources. The first source came from the main IDIs conducted with fathers, mothers, and community stakeholders. Fathers were asked to describe their daily routines, time spent with their partner and child, and perceived barriers and enablers to caregiving (e.g., “*Please describe how you spend your time during a typical day*”; “*What makes it difficult to spend time with your young child?*”). Mothers were asked parallel questions about their own routines and their perceptions of how their partners spent their time. These narratives enabled us to examine how fathers’ time use was understood from both their own perspective and their partner’s. Similar questions on how parents in the community spend their day and barriers and enablers for parents’ time spent with their family were asked from community stakeholders, who provide an external perspective on these issues. IDIs typically lasted approximately 60–90 minutes while FGDs were slightly longer at around 90 minutes total.

The full sample for the original parent study included 120 respondents: 56 fathers, 56 mothers, 3 community health workers (CHWs), and 5 community leaders. This sample size was reached based on thematic saturation for the primary research aim of the broader study, which sought to understand fathers’ roles in the lives of young children. Saturation was assessed through ongoing team debriefs and review of interview content, and was determined when no new themes or subthemes were emerging across the core domains of caregiving roles and responsibilities, couple relationships, and parental stress and mental health. The present analysis represents a secondary exploration of fathers’ time use, drawing on the same set of interviews to examine how fathers spend their time and what factors shape that time use within the context of parenting.

The second source of time use data came from a subsample of fathers who participated in a follow-up interview 1–3 days after their initial IDI during which they were asked to retrospectively recount how they spent their time and complete a diary log (S1 File). During these follow-up sessions, research assistants asked fathers to think through all their activities over the previous days in detail and break down each activity by time, duration, location, as well as identifying the individuals they were with at each time point. The research assistant then recorded this information on a paper diary log. In addition to documenting this information, the research assistant asked follow-up questions about why fathers engaged in particular activities and probed specifically about whether their partner or child accompanied them during different activities and time periods. When fathers discussed childcare, we specifically probed about their involvement with the child under 2 years of age. Fathers were also asked if the past two days reflected their typical routine or included any unusual event. These qualitative narratives provided contextual information, including motivations, social interactions, and the typicality of activities, that would have been difficult to capture in a purely quantitative time-use survey.

The outcome of this exercise was a completed paper diary log and a recorded narrative interview. The audio recordings were later transcribed and translated, providing richer content for interpreting the diary logs. The structured paper diary log was used to systematically capture fathers’ daily schedules in a standardized format, while the accompanying qualitative interviews provided additional narrative detail on the context and meaning of each activity, including motivations, social interactions, and whether the activities reflected typical routines. These follow-up time use IDIs lasted approximately 30–45 minutes.

Among the 56 fathers who participated in the original IDIs, 14 were selected and agreed to participate in the time use diary interviews. This subsample was not intended to achieve thematic saturation, but rather to generate illustrative, in-depth accounts of paternal time use and complement the broader data through triangulation.

### Data analysis

After completing the initial coding in the parent study, we conducted thematic content analysis to identify themes about how fathers spend their time in a typical day, as well as the barriers and facilitators of their time use and particularly regarding childcare. Our analysis triangulated data from three sources: (1) in-depth interviews (IDIs) with fathers and mothers; (2) focus group discussions (FGDs) with mothers, fathers, and mixed-parent groups; and (3) time use diary logs and their accompanying IDIs with a subsample of 14 fathers. Interviews and discussions were audio-recorded, transcribed, and translated into English by experienced Tanzanian translators. Using Atlas.ti, three research analysts independently coded the English transcripts from the IDIs and FGDs, with 30% of the transcripts randomly selected to be independently coded by a second analyst [33]. Emerging themes and any discrepancies during coding were then discussed during weekly meetings with the study team.

For the time diary interviews, two research analysts reviewed the structured diary logs and narrative transcripts to create a detailed profile for each participating father. These diary logs included descriptions of fathers’ activities at each hour throughout the day, the location and duration of each activity, any other individuals who were present, and any exemplar quotes. Exemplar quotes were selected when fathers provided particularly rich or detailed descriptions of specific activities, explaining not only what they were doing but also why, with whom, and how those activities fit into their broader daily routines or family dynamics. Themes and patterns from these diary logs were used to triangulate the findings from original IDIs and FGDs and develop a more detailed understanding of how fathers spend a typical day. For example, we compared the general descriptions of childcare routines from the IDIs with the time-specific accounts in the diary logs (e.g., feeding or playing with a child before leaving for work in the morning) to assess whether fathers’ patterns of involvement aligned across data sources. We also purposefully included quotes from mothers and community stakeholders to present diverse perspectives on fathers’ time use in parenting, ensuring that both household parenting experiences and community-level practices informed our interpretation of the findings. Throughout the analysis, the study team held regular debriefs and reviewed preliminary findings collaboratively. All thematic results were further validated by two other research analysts on the team.

### Ethics approvals

The protocol for this study was approved by the Institutional Review Board of Harvard T.H. Chan School of Public Health (IRB22–0235) and the National Institute for Medical Research in Tanzania (NIMR/HQ/R.8a/Vol.IX/4076). All participants included in this study provided their written informed consent. The consent forms were administered aloud in Kiswahili by the research assistants who were conducting the interviews, and all participants were provided with the opportunity to ask any clarifying questions.

## Results

The results are organized into three sections. First, we describe fathers’ daily routines, highlighting work-related and non-work-related activities. Second, we identify key barriers to paternal involvement in parenting, including long working hours, physical exhaustion, and gender norms. Third, we identify factors that facilitate father engagement, such as flexible work options, more equitable attitudes, and a desire to build closer bonds with their children. We observed notable differences in time-use patterns by occupation (farming versus fishing), and these distinctions were presented in the first section. However, barriers and facilitators to paternal engagement were largely consistent across occupations and are therefore presented overall in the second and third sections.

The mean age of fathers was 36 years (standard deviation [SD]: 9), and the mean age of mothers was 27 years (SD: 6). Sixty-six percent of fathers and 80% of mothers had completed primary school as their highest level of education. All caregivers were married. All community leaders were male, with a mean age of 45 (SD: 16), while the majority of CHWs were female, with a mean age of 44 years (SD: 12). More detailed socioeconomic information is presented in the parent study [31].

Overall, fathers spent the majority of their day out of the home working or seeking employment opportunities. During a typical day, fathers left home early in the morning after sunrise and did not return until the evening or later at night, only stopping at home briefly for lunch and a period of rest midday. In addition to spending most of their days on income-generating activities, many fathers spent their afternoon and/or evenings socializing with friends. Poverty both at the household level but also structurally in terms of community unemployment and lack of formal jobs largely contributed to fathers spending most of their time out of the home in search of living wages. All fathers expressed the importance of but also pressure to financially provide for the household, with families largely dependent on the fathers’ contributions. See Table 1 for two illustrative case examples of how fathers in our study sample spent their time during a typical day.

**Table 1:** Diary log of fathers’ time use during a typical day.

Time	Example father A: farming as primary livelihood	Example father B: fishing as primary livelihood
6:00	Wake up	Wake up
7:00	Goes to farm and water crops	Have tea
8:00		Goes to work to repair small fishing boats
9:00		
10:00		
11:00	Finishes watering crops and returns home. Eats lunch at home	
12:00	Goes to community center to socialize with friends	
13:00		
14:00		Returns home for lunch and time spent with wife and child
15:00	Returns home for a meal. Then goes to farm again	Returns back to work to continue labor
16:00	Waters crops again	
17:00		
18:00	Finishes watering crops and goes to community center	Returns home and spend time with child
19:00	Watches football with friends	Socializes with friends and listens to news at community center
20:00		
21:00	Returns home. Eats dinner	Returns home. Eats dinner
22:00	Goes to sleep	Goes to sleep

Note: Shaded cells indicate time spent out of home; unshaded cells indicate time spent at home.

### Fathers’ daily schedules were dominated by work, with patterns varying by type of employment and seasonality

All fathers noted work as the primary activity of their day. Fathers most often engaged in farming, fishing, and small business, with many participating in multiple types of work (e.g., both farming and fishing, both farming and selling milk). Less common jobs included carpentry, casual labor, or motorbike drivers. While fathers’ time patterns were largely generalizable across men in our sample, employment type explained some variability in fathers’ schedules.

Fathers whose primary work was farming had more routine schedules relative to other types of employment. Farmers typically woke around 6am or earlier and left their homes between 6am to 9am to work in the fields until midday. Many farmers focused on checking their crops, weeding, or working on the irrigation systems of their farm while some farmers travelled to other cities to sell their produce. Fathers often went to farm with other companions, such as partners, other family members (e.g., in-laws, older aged children), and neighbors, to work together or receive assistance. In addition to field work, some farmers also spent time in the mornings feeding cattle or grazing fields for their cows. After completing the bulk of their agriculture work in the mornings, nearly all fathers returned home for lunch and to relax and rest for a few hours. After lunch, some fathers returned to the field to continue farming activities while others visited the town center to watch sports and socialize. For example, one father described his typical day as follows: *“My daily routine from morning I wake up at 6am and go to the farm until 9am, then I come back and go to feed cattle, at 1pm I come back at home, at 3pm I go to the farm again to water the crops and then I come back home. That is my time.” (Father IDI, village 1, #14)* Several fathers who engaged in agriculture work highlighted the seasonality of this work and how, for example, to prepare for the harvest during the rainy seasons, the activities and time spent working in the fields increased (e.g., farming in multiple fields). In some households, both parents engaged in farming. For example, one father spent the early afternoon at home with his wife and child, until both parents left to water their crops during the evening.

Fishing was the second most common type of work among fathers. While there was more variation in the time patterns of fathers who engaged in fishing, most men spent longer durations of time out of home and at their workplace than farmers. Some fishermen had similar schedules to farmers: leaving home early in the morning, returning home to rest and eat in the late afternoon, possibly going back out to fish, and returning home by night. However, other fishermen frequently worked overnight by lakeshores for multiple days or up to a few months before returning home. For example, referring to her husband’s work schedule, one mother said, *“There is a time when they [fishermen] go and sleep in the lake and then they come back, they can go there today, spend four or five days and then come back home.” (Mother IDI, village 1, #1)* Although the specific work activities differed for each fisherman, tasks typically included catching, buying, and selling fish, making, repairing, and organizing fishing nets, and repairing boats of their own or for customers.

Other income-generating activities for fathers included working in small businesses like libraries or furniture stores, driving motorbikes, and seeking daily wage labor. Many of these jobs were often associated with considerable time away from home, especially during the evenings, and more inconsistencies in daily schedules compared to farming or fishing. For example, some fathers who sold milk for work mentioned doing so in the late afternoons and evenings after they collected milk in the mornings: *“[Work] responsibilities are to be at the farm in the morning for food cultivation and in the afternoon coming back to wash milk containers and getting ready to distribute milk. And after taking milk, I don’t come back [home] until evening.” (Father IDI, village 1, #13)* Like some farmers, these fathers often travelled to sell their product in surrounding towns or delivered directly to people’s homes. Motorbike drivers also appeared to work frequently in the evenings when business was often busiest. One father who worked in construction shared how the specific days of his construction work were irregular. On days when he was called for construction work, he would leave home early in the morning and return home at the end of the day in the evening. However, on most days where there was no such work, he stayed largely at home.

### Despite long work hours, fathers found time for childcare and family interactions, though participation varied widely

While work-related activities consumed most of fathers’ daily time use, at least half of fathers also mentioned engaging in non-work activities as part of their typical routines, including parenting and childcare, family interactions, and leisure. Fathers’ shared time with their families largely occurred in the early mornings at home before fathers left for work, midday when fathers returned home for lunch, in the evenings after work, or during weekends when fathers commonly rested from work and spent more of the daytime at home. At the same time, several other fathers expressed limited engagement, or even no time at all, for childcare or household chores due to their busy work schedules. *“I spend a lot of my time working in order to get income for supporting my family as a result, I have little time with my partner and Jordan.” (Father IDI, village 1, #6)*

In the early mornings before leaving for work, some fathers described helping with childcare or household chores (e.g., playing with children, fetching water). When describing his typical day, one father noted how he cleaned the family compound and washed clothes before leaving for work, *“I wake up at 06:00Hrs, I clean house surroundings and do other small activities such as washing clothes. After that I go to work from 09:00Hrs until 12:00Hrs then I come back home for lunch.” (Father IDI, village 4, #1)* In addition to early morning before leaving for work, many fathers mentioned mid-afternoon when they returned for lunch as another moment when they engaged in non-work activities, such as conversing with their wives and/or children and relaxing before returning to work. For example, one father described how he typically conversed with his wife during lunchtime about life generally as well as how the child was developing.

After work in the evenings, nearly all fathers mentioned socializing with other men in the community in different locations in the center of the village. Many fathers reported watching football with friends and other members of the community: “*If I finish at work, sometimes if there is a football challenge most of the time I go to watch football game, so in most cases the time to arrive at my home is always at night.” (Father IDI, village 1, #8)* Several fathers mentioned discussing work-related challenges or providing social support to their peers during these social gatherings. Fathers usually returned home around 7 pm or later at night. If their child was still awake, some fathers played with them while their wives cooked. For example, one father shared, *“Jordan is both our responsibility, when I come home, I carry him and play with him while his mother is preparing food for us.” (Father IDI, village 1, #6)* After dinner, some fathers sat with their wives and/or children and discussed how they spent their days. Most fathers went to bed between 8 pm and 10 pm.

During weekends, fathers spent more time relaxing at home and engaging in other non-work activities. Most fathers mentioned going to markets or attending church with their families on the weekends. Many fathers highlighted having more time to spend with their partners and children as well as to visit other relatives and family members, including siblings and in-laws. During these visits, they discussed various topics, such as life matters and how to take care of their children. Fathers were also more likely to engage in childcare activities during the weekends, with one father mentioning taking his child for a walk: *“Yesterday [Sunday] after breakfast, I took him for a walk and then I returned him to his mother to play.” (Father IDI, village 1, #3)* However, some fathers were unable to spend time with their children even on weekends, depending on the nature of their work. For example, some fathers mentioned farming all days of the week even on the weekends and that constantly occupying his time.

### Long work hours, exhaustion, and gender norms limited fathers’ time at home and engagement in childcare

Fathers described multiple barriers to spending time at home and on childcare, including long work hours to provide for their family, extended periods away from home for work, exhaustion due to work, and restrictive gender norms. Many fathers perceived their breadwinning roles as the most important paternal responsibility, which explained their limited engagement in childcare or household activities. One father articulated, *“Fathers in our society are responsible for taking care of the family; that makes their time at home limited but, our wives are at home for the most of time which makes them in a good position to play with children and look after them. I may just go back at home for lunch and come back to work. I would like to play with children, but I don’t have enough time.” (Mixed FGD, village 5)* Fathers consistently underscored the conditions of poverty, the opportunity costs of not working, and the need to secure money each day as the primary reason for prioritizing and valuing work above all other activities. For example, one father and a mother shared:

*“Fathers spend most of the time at work in order to provide for the family. If a man, instead of going to work, he takes the child to the clinic while his partner stays at home, how will this family survive for this day? Where will they get the food? It’s not that we have shifted that burden on them [women], we just have to work in order to provide for the family while they fulfill [childcare] responsibilities.” (Father IDI, village 1, #4)*

*“There is nothing we can do [to get more time to spend with the partner] because he must work in order for us to get food, what are we going to eat if he decides to stay at home?” (Mother IDI, village 2, #5)*

Many also highlighted the lack of formal and stable employment opportunities in their community that necessitated fathers to work long hours whenever business was available or to be available to work at any time given the precarious nature of work. Poverty and limited employment further contributed to fathers feeling limited agency or control over their time availabilities and schedules. A few fathers even believed that it was not critical for men to spend time engaging in childcare activities, like playing or communicating with the child, as long as they were working hard to financially provide.

Several parents noted that because fathers spent most of their day out of the home looking for the work, by the time they returned home it was often late at night and their children were already asleep. For instance, one mother shared, “*I think it is because of his work, he doesn’t get enough time to be with his child, sometimes he finds her already asleep and when he goes out for work she may be still asleep.” (Mother IDI, village 2, #7)* As another example, one father explained: “*In this community, mothers are the main caregivers to children 0 to 3 years old because fathers are busy. A father comes back at night when a child is already sleeping. And will only know about the child’s progress by only asking from mothers and not seeing the children by themselves.*” *(Father FGD, village 3)*

Certain jobs appeared to have greater restrictions on men’s time use and further limit their engagement in caregiving activities with their children and partners. Fathers working in fishing reported spending extended periods of time away from home, while those in agriculture were closer to home and therefore tended to make frequent visits to home, even when busy. One father who worked as a fisherman shared, *“I am involved in fishing activities in which I spend the entire day during this season when fish are scarce, due to that, I don’t have enough time to spend with my child and family.” (Father IDI, village 1, #6*) Another father who was in the industry of selling fish also noted how this work occurred more in the evenings, *“For example, on [selling] fish it is a work that its time is openly known at evening 1600 so I will have more time with my family in the morning.” (Father IDI, village 1, #7)* However, at the same time, fathers engaged in agriculture also noted how seasonality influenced their time availability, with time constraints increasing during the harvest season: *“In this rainy season, I do not go to the farm for watering vegetables so I have more time be at home most of the time, unless I go out to buy family needs.” (Father IDI, village 4, #11*)

Exhaustion from working all day was also noted by many fathers as preventing them from engaging in other activities when they returned home, despite having the time. A few fathers described not having the energy to hold or play with their child or converse with their partners upon arriving home. For example, one father shared, *“A man has to go to work from 06 am to 8pm and they come home tired. You cannot get time to go near a child and when a child comes near you, you act unfriendly because you are tired. Even the communication with the wife becomes poor, you are angry all the time.” (Father FGD, village 2)* A community leader highlighted how stress and failure in earning enough money also affected fathers’ mood and wellbeing and his levels of family engagement when returning home, *“A big thing are financial issues. Fathers go to work and come back very tired, or if he comes back from his business with less [money] then we will be with a lot of frustrations and not speak to anyone.” (Community leader IDI, village 1)*

Many fathers valued leisure time with friends in their free time given the exhaustion and stress caused by their work. But these activities, such as socializing with friends at the village center or watching sports, were often in the evenings and then further contributed to men returning home late. A few fathers and community stakeholders mentioned that some fathers engaged in excessive alcohol consumption in these social contexts, which further drove fathers away from their families or resulted in fathers’ money spending money to support their drinking habits rather than to provide for the family. For example, one community health worker shared:

*“They usually leave at 0700Hrs and come back at 1400Hrs, if they didn’t earn anything at that day they come back home with nothing, and if they got some money they may go to drink alcohol and return at home at night asking for food while they didn’t leave anything at home, where does his wife get the food from?” (Community health worker IDI, village 3)*

A community leader in another village similarly noted alcohol use as a barrier to fathers’ engagement in childcare: *“Another reason can be alcohol, when you come home from work drunk and straight to bed you will never get a chance to play with a child because you leave home while they are still sleeping but if as a father you come home early, you will play and talk to a child.” (Community leader IDI, village 2)*

Finally, restrictive gender norms were another underlying barrier to fathers’ values and priorities in how they managed and allocated their time. Fathers equated earning money and a being a breadwinner as an indicator of their masculinity and failing to do so was stigmatized and viewed as being less than a man: *“A man has to provide food for his family… if he does not support his family, he is weak.” (Father IDI, village 2, #5)* Several community stakeholders suggested additional restrictive gender norms, which they referred to as “kingship behavior”, or the view held by some fathers that regardless of their availability childcare activities were not part of their expected roles and instead a primary responsibility of women. For example, one community leader shared: *“While some don’t have time, others they have a certain kingship behavior of not sitting and talking with their children.” (Community leader IDI, village 1).*

### Flexible work, equitable attitudes, and a desire for close father–child bonds motivated fathers to spend more time at home

Several facilitators enabled fathers to spend more time on childcare and with their family including autonomy and control over their work schedule, more equitable gender attitudes, and the personal desire to strengthen father-child bonds. Fathers shared how having more agency in their work, such as through owning their business or having the resources to complete their work more efficiently, would allow them to spend more time at home. For example, one father shared, *“If I can get enough capital to keep cattle at home or open a big shop of selling crops at home, these could help me to be at home every day and hence get enough time with my family.” (Father IDI, village 4, #12)* Furthermore, fathers mentioned that having a stable job, with a fixed work schedule, would allow for more consistent time spent with their child: *“If I get a formal job that will help me meet family needs, food, shelter and everything. I will be sure of when I wake up and work, what time I spend with my children and everything but now I don’t know when I will be at home and when I will be out working, when I get a job I leave no matter what is the time.” (Father IDI, village 2, #4)* Additionally, one father believed that if mothers additionally carried out income generating activities, such as by running a small business, he would be able to spend more time with his child, *“Maybe if I get capital to initiate a business even here at home it can be very helpful because even my wife can do it and me as a father I can remain at home with my child.” (Father IDI, village 4, #7)*

Fathers who held more gender equitable attitudes by viewing the role of fathers as a nurturer and supportive partner, rather than solely as a provider, were more likely to personally prioritize family time in their schedules. One mother shared how her partner would be more likely to spend time with his family if he viewed that as a critical part of his responsibilities within the family, *“It could help if he would feel the need for us to get time and talk. Then he would spare more time for us.” (Mother IDI, village 1, #8)* While few fathers described communicating with their partner throughout the day about household finances and livelihood decisions, such behaviors were evident among fathers who held more positive family values. For example, one father who regularly played with his child and described having a loving, supportive relationship with his wife mentioned if he had more time he would spend it talking with and advising his partner, “*If I had more time, I would use it to deliberate and discuss with her, to advise each other on caregiving for our children, keeping our children and to be near our children so make them to be mentally strong.” (Father IDI, village 4, #1)*

Fathers who were already engaged in household chores and perceived this as part of their family responsibilities were also more likely to make time to engage in parenting and childcare activities. These activities included teaching children to read and reading books with them, taking children out of the home with them, displaying affection, and playing with them. For example, one father who was already engaged in many household chores expressed a positive attitude and desire to play more with his child if he had the time, “*I would play more with her, carry her and clean her up [if I had more time]. For example, skipping a rope, playing with a ball, and showing her different things. For example, I was helping her with cleaning the surroundings before I came for this interview today.” (Father IDI, village 1, #1)*

Finally, a few fathers were motivated to spend time with their young child to strengthen their father-child bond. For example, one father shared,

*“I want to use whatever time for my child. I will be very happy by looking at my child, and he will also be happy by knowing that my father is available and carrying her. If you are not available the child may forget you, the children sometimes may cry and deny the father because you have taken a long time to know the child. But if you are close to him, the child knows you early as the father.” (Father IDI, village 1, #7)*

## Discussion

This study aimed to contextualize fathers’ time use and identify the factors influencing their time allocation and especially towards parenting. Our findings reveal how fathers mainly allocate their time towards work and searching for income-generating opportunities, but also make time for parenting and leisure activities throughout their days. Overall, these results shed light on men’s time patterns and the time constraints they face, providing information that can help identify specific times and contexts within their daily routines where efforts to increase father engagement in parenting could be effective.

Fathers’ work was the most time-consuming activity of their days that drove men to spend the majority of daytime hours outside of the home. Moreover, fathers valued work as the most important responsibility of fatherhood and thus most deserving of their time and energy. Consequently, they perceived a direct tension between their parenting responsibilities, which would require them to mainly be at home, versus their roles as financial providers for their family. These time constraints were influenced by the nature of employment opportunities in the study context. Many fathers were involved in small businesses or casual jobs with unpredictable and inflexible work schedules, posing challenges in balancing their work commitments with parenting responsibilities.

Both fathers and mothers recognized significant opportunity costs when fathers allocate less time to work in order to contribute more to child and family caregiving activities. This finding has been underscored in several other studies in LMICs, including in East Africa. For example, fathers often cited these opportunities costs as the primary reason why they could not accompany mothers to health facilities for child healthcare seeking in a study in rural Mozambique [17] or had low participation rates in a community-based parenting interventions in rural Kenya [34]. In another example in rural Uganda, retention of fathers in a parenting program was higher among fishermen compared to workers in other job types such as construction or cleaning, as fishermen had more regular work schedules, enabling their participation in interventions [35]. These results reinforce how work responsibilities and even occupation types play a key role in influencing fathers’ time allocation [31,36] and father-child relationship quality [37]. Recognizing this, previous father-engaging interventions in high-income settings, such as the Early Head Start Program in the U.S., were designed with a multi-component approach, combining employment services and job skills training with parenting support. These integrated strategies aligned with fathers’ economic priorities, potentially increasing their engagement [38].

Secondly, the priority and expectation around fathers’ time spent on work was closely intertwined with prevailing patriarchal gender norms, which reinforces values of men’s power and expectations as the breadwinner for the household [22,39]. This norm often precipitated pressure to provide and further contributed to fathers working even longer hours, resulting in physical exhaustion and mental stress that limited engagement in parenting responsibilities by the time men returned home [31]. Even if fathers were not employed or working, they were constantly looking for available work outside the home in hoping for securing more income-generating opportunities to live up to these gender expectations and ideals. For example, in another study from South Africa, fathers not only felt pressured to meet the basic needs of their family members but also mentioned expectations from their children to bring food and gifts, reinforcing traditional norms of men as providers [38].

Furthermore, it is important to recognize that father’s time use towards work activities is correlated with structural factors, including poverty, low economic development, precarious job markets, and increased food insecurity exacerbated after the Covid-19 pandemic. The Tanzania National Panel Survey indicated a slight increase in the poverty rate, coinciding with the rise in global food prices due to these circumstances [40]. These structural barriers can further increase the opportunity cost of fathers’ time for parenting, while also exacerbating financial stress and fear of not conforming to community norms surrounding fatherhood. Similarly, a study in rural Mexico found that the introduction of mechanized farming and the resulting improvements in economic conditions led to increased parental investment in childcare [41]. This highlights how fathers’ time use patterns can be influenced by broader market conditions and economic development.

While fathers spent time primarily working during the day, we found that in the evenings they commonly socialized with friends and other members within their communities. Specifically, fathers often met with others to watch sports or discuss daily events or challenges. These distributions of fathers across work and social events have been shown in population-level studies. For example, in Ghana and South Africa, fathers reported an average of five hours for leisure and social activities, slightly less than the time devoted to work [42]. Fathers’ time spent on social and leisure activities were more frequently noted during weekends when they noted that they had more time availability from being less busy with work.

Even though fathers spent most of the day outside of the home for work or socializing, still many fathers also engaged in parenting roles and interactions with their young children and partners at home during the relatively brief moments they were at home. These activities encompassed routine household chores such as cleaning the house and fetching water, direct interactions with their children, such as playing, taking them out for a walk, or communicating with them, the provision of emotional or practical support to their partners or discussing family issues and making decision together. Fathers were more likely to engage in these parenting and childcare activities when they came home for mealtime and on the weekends when they spent more time at home or often went with their partners and children as a family to visit other relatives. We found that strong family values, positive couples’ relationships, and fathers’ desires to strengthen their bond with their children were major facilitators of fathers’ time spent towards parenting. Similar findings have been described in studies from rural South Africa [43] and Madagascar [44], where in spite of prevailing restrictive gender norms, fathers’ attitudes are shifting with men desiring to build nurturing relationships with both their children and partners. These findings indicate that fathers’ priorities do not solely revolve around either work or parenting in a mutually exclusive manner. Instead, key facilitators can be leveraged to increase their time spent at home with their family.

Our results have several applications to the design and delivery of parenting interventions that seek to engage and support father involvement in family caregiving [18]. In terms of timing, implementers should consider scheduling interventions during specific times of the day, weekends, or post-harvest seasons when fathers have greater control over their time. For instance, many fathers returned home for lunch despite spending the majority of their day out for work, which highlights a specific moment during the day to strengthen father-child relationships. Weekends and evenings are also key windows when fathers spend extended periods of time at home. Targeting these moments, when men are already commonly at home and often physically together with their children and partners, may foster greater father participation and more involved fatherhood. In addition, offering flexibility in the location and types of interventions has been effective. For example, the Family Foundations program leveraged institutional niches by providing parenting classes through childbirth education departments at local hospitals [18,45].

Our results also revealed some insights about fathers’ social networks and how fathers also commonly spent time with their peers. Fathers valued meeting others in the community after work to relieve their daily life stressors and gain social support from their peers. Leveraging these existing social networks and locations to fulfill these purposes may be an effective way for reaching and engaging fathers. Such programs can create a safe and open environment for men to discuss sensitive issues with their peers and the come up with action plans to address their psychosocial challenges and caregiving aspirations [35,46]. Additionally, targeting intervention activities in locations where fathers typically spend their leisure time on weekdays and weekends, such as village centers, churches, or markets where men are already meeting, could be more successful than home visits or setting up separate gathering spaces for fathers.

Furthermore, to facilitate changes in men’s time use and promote more engaged fathering, it is recommended to develop a comprehensive parenting program that encompasses norm change at community level. Such a program should create an open platform for understanding and sharing the concept of fatherhood and what constitutes a good father within the community [47], taking into account the multifaceted roles that fathers balance with, including economic provision and psychosocial support. For example, a study in rural Tanzania found that fathers showed greater acceptance of gender-equitable norms and increased engagement in stimulation when couples participated jointly in a parenting intervention, compared to a mothers-only intervention. The couples intervention included components on positive masculinity, gender norms, healthy relationships, and parenting [48,49]. To address fathers’ caregiving experiences in Mwanza holistically, parenting programs should incorporate components that can help address fathers’ financial stress and ideally also provide opportunities for income generation. This may involve training for new skills, providing financial support, or supporting pilot businesses to create new job opportunities. By addressing economic empowerment alongside parenting interventions, fathers can be better equipped to fulfill their roles as active caregivers while also contributing to their families’ financial stability [50].

Our study presents novel qualitative data on fathers’ time use patterns from the perspective of fathers and further triangulated with that of mothers and other community members. Our results shed light on strategies to shift fathers’ time use towards parenting and through the design and implementation of parenting programs in the context of Mwanza, Tanzania. Importantly, the diversity we showed in fathers’ time-use patterns highlights the value of designing strategies that are flexible and tailored to the distinct schedules and constraints faced by different groups of fathers.

However, there are some limitations to consider in this study. Firstly, the sample focused on fathers who specifically co-resided with their families, and so we may not have captured the full range of time use patterns among other types of fathers such as separated or single fathers. Additionally, because our analysis focused on fathers’ parenting of children under the age of two, we were unable to examine in detail how fathers allocate their time among children of different ages within the same household. Future research exploring family structure and related dynamics would offer a more nuanced understanding of how fathers allocate their time. Also, as this study was a qualitative investigation, we did not quantify the number of hours that fathers devoted to specific activities during the day. Therefore, we cannot make direct comparisons of fathers’ time use across different activities. Lastly, the study’s findings primarily reflect the time use patterns observed in the specific context of Mwanza, which may limit the generalizability of the results to other regions within Tanzania or other countries.

## Conclusion

In this qualitative study, we contextualized fathers’ time use in their daily lives and identified the perceived barriers and facilitators to their engagement specifically in fathers’ family caregiving activities. Employment, poverty, financial stress, and restrictive gender norms were factors that constrained fathers’ time availability and parental engagement at home with their children. Through examining how fathers allocated their time, we gained new insights and ideas for where, when, and with whom fathers could be reached in efforts to get men more engaged in parenting. Future parenting programs should be designed not only recognizing fathers’ daily routines in the given context, but also practically in a way that enables men to invest more time with their young children, partners, and families to ultimately increase gender equity and improve child development.

## Supporting information

S1 File Format of time log diary. (DOCX)

S2 File Inclusivity in global research questionnaire. (DOCX)

## Abbreviations

FGDs: focus group discussions
IDIs: in-depth interviews
LMICs: low- and middle-income countries
