# Supplementary material for: Outside versus inside the home: Tensions between fathers’ work and parenting responsibilities in Tanzania
Source: PLoS One. 2026 Jan 29;21(1):e0341670. doi: 10.1371/journal.pone.0341670 (PMC12854416; doi:10.1371/journal.pone.0341670)
Supplement: S1 File — (DOCX) [file pone.0341670.s001.docx]

**Supplementary Online Appendix: Format of Time Log Diary**

Father’s name: ____________________________ Research assistant’s name: ______________________

Date of interview: ______________________

*Please complete in English*

| *Method: Completed together with participant and RA on paper-based form* | | | | *Method: RA probed to better understand the activity (audio recorded)* | | |
| --- | --- | --- | --- | --- | --- | --- |
| Date | Activity | Start – end time (AM/PM) (Military time) | Setting/location | Why did you go there/why did you do this activity? | What did you do at this location/during this activity? | With whom did you interact with during this activity? (Probe on partner and child) |
|  |  |  |  |  |  |  |
|  |  |  |  |  |  |  |
|  |  |  |  |  |  |  |
|  |  |  |  |  |  |  |
